# Supplementary material for: Topological basis of signal integration in the transcriptional-regulatory network of the yeast, Saccharomyces cerevisiae
Source: BMC Bioinformatics. 2006 Oct 28;7:478. doi: 10.1186/1471-2105-7-478 (PMC1643839; doi:10.1186/1471-2105-7-478)
Supplement: Additional file 1 — Supplementary Material. Detailed Methods, Supplementary Table, Supplementary Figures. [file 1471-2105-7-478-S1.pdf]

Supplementary Material

Topological basis of signal integration in the transcriptional-regulatory network of the yeast, *Saccharomyces cerevisiae*

Illés J Farkas[1,2]*, Chuang Wu [3]*, Chakra Chennubhotla [3], Ivet Bahar [3], Zoltán N Oltvai [1§]

[1]Department of Pathology, University of Pittsburgh, Pittsburgh, PA, 15261, USA
[2]Department of Biological Physics and HAS Group, Eötvös University, Budapest, 1117, Hungary
[3]Department of Computational Biology, University of Pittsburgh, Pittsburgh, PA, 15261, USA

Contents

Detailed Methods 2

Supplementary Table 5

Supplementary Figures 6

References 14

Detailed Methods

Databases and Software

The publicly available dataset on the TR network of *Saccharomyces cerevisiae* was downloaded from the supporting website of the original publication (Ref. [1] http://jura.wi.mit.edu/fraenkel/download/release_v24). This computationally filtered dataset, originally obtained in rich media and a few other growth conditions, lists directed binary interactions at various confidence levels, and is further improved by including additional transcriptional interactions from the literature [1]. All computational analyses were performed with the SGD IDs of the genes that were then transformed back to traditional gene names for easier presentation. Conversion tables were downloaded from the Saccharomyces Genome Database (SGD, http://www.yeastgenome.org) and the MIPS Comprehensive Yeast Genome Database (CYGD, http://mips.gsf.de/genre/proj/yeast/). Of the six different datasets representing various confidence levels [1], we used the highest confidence data set for most of our analyses (Supplementary Table S1). Originally, the network derived from this dataset contained 1905 genes and 3406 regulatory interactions, which we reduced to 1905 nodes and 3394 directed links by removing 12 autoregulatory links. The resulting network contained 99 TFs (54 input and 45 intermediate nodes) and except for two small isolated groups – with the input nodes Pdr3 (drug resistance, regulating itself and one other gene) and Zap1 (zinc-regulated, regulating four other genes) - it is comprised of one giant connected component. Most targets (intermediate and output nodes) are regulated by more than one (on the average, 1.8) TFs. We quantify the relative overlap between the target lists (A_i and A_j) of two TFs (*i* and *j*) by the Jaccard correlation, $\left|A_i \cap A_j\right| / \left|A_i \cup A_j\right|$, between the two sets. An alternative representation of the TR network is to consider only TFs and the regulatory interactions between them, in which case the network contains 99 nodes of which 69 are connected in a giant component.

The normalized microarray expression data sets GDS18-20, GDS112-115, and GDS362 were downloaded from the FTP directory of NCBI's Gene Expression Omnibus (GEO) at ftp://ftp.ncbi.nih.gov/pub/geo/data/gds/soft_gz. Our programs were written in Perl and C++, and for visualization we used the Linux tools Xfig and Gnuplot together with the network drawing program Pajek [2].

Network randomization and graph motifs

To assess the enrichment of 3-node subgraphs in the regulatory network, we used link randomization tests [3] that preserve the number of incoming and outgoing links around each node, but obliterate all other information about the connectivity of the network. In one step of this method two links, A→B and C→D, are selected randomly and moved to the unoccupied A→D and C→B positions. We examined n_N = 100 randomized networks, each produced with n_S = 100,000 rewiring steps starting from the original TR network, i.e., each link was moved approximately 60 times to generate a given randomized network. Following Ref.

(3) a subgraph with M_0 copies in the original TR network and $M \pm \Delta M$ copies in the randomized versions is called a graph *motif*, provided that the associated Z score, $Z = (M_0 - M) / \Delta M$, is significantly positive. We also verified that for the TR network studied here n_N and n_S are both sufficiently large to ensure the convergence of the Z-scores for 3-node subgraphs.

Cumulative GO categories

For functional characterization of yeast proteins we grouped the 33 Gene Ontology (GO) Slim Biological Process terms [4] into the following eight categories: *cell cycle*-related (GO terms: cell cycle, cell budding, conjugation, cytokinesis, meiosis, pseudohyphal growth, sporulation), *metabolism*-related (GO terms: amino acid and derivative metabolism, carbohydrate metabolism, cellular respiration, DNA metabolism, generation of precursor metabolites and energy, lipid metabolism, protein catabolism, RNA metabolism, vitamin metabolism), *morphogenesis*-related (GO terms: cell wall organization and biogenesis, cytoskeleton organization and biogenesis, membrane organization and biogenesis, morphogenesis, nuclear organization and biogenesis, organelle organization and biogenesis, ribosome biogenesis and assembly), *transcription and protein synthesis*-related (GO terms: protein biosynthesis, protein modification, transcription), *transport*-related (GO terms: electron transport, transport, vesicle-mediated transport), *stress and homeostasis*-related (GO terms: cell homeostasis, response to stress, signal transduction), *cell movement*-related (GO terms: substrate-bound cell migration and cell extension), *unknown* (biological_process, biological_process unknown, unknown), respectively.

Task integration by overlapping origons

A simplifying view of the TR network is provided by the origon representation [5], shown by color-coded circles in Figure 1*B*. Each origon represents a cluster of nodes originating from a common (input) TF (54 of them in the present case), and the color code therein describes the occurrence of four types of interaction motifs distinguished by their high Z-scores (see below). Except for the two input nodes mentioned above (Prd3 and Zap1), all origons are interconnected due to the partial overlaps between their members at intermediate and output layers. The number of shared members is reflected by the thickness of the links between the origons. The examined yeast TR network has 418 such overlapping pairs of origons.

Of interest is to characterize the degree of integration of functional tasks between overlapping pairs of origons. To this aim, we first removed from the TR network all genes with GO Slim annotation unknown, and counted the number of genes annotated by a given GO Slim term, within the subsets A^B (overlap), A\B and B\A (genes contained only by A or B) for each pair of overlapping origons (A. B). Three vectors, defined by the fractions/probabilities of GO slim terms were thus generated for each pair, denoted as ***a*** (for A\B), ***b*** (for B\A), or ***c*** (for A^B). The overlap (A^B) integrates tasks from the other two regions, if ***c*** is sufficiently similar to both ***a*** and ***b***. The extent of similarity between the three probability distributions was then assessed by the correlation cosines (***c* . *a***) and (***c* . *b***),

expressed by the sum K = ***c* .** (***a***+***b***), where the dot designates the scalar product. We found that the K values for pairs of origons in the yeast TR network were significantly higher than those calculated for 100 randomized test cases. The corresponding Z score – i.e. (<original K value>–<average K in random cases>) /<standard deviation in random cases> – averaged over all pairs was <Z(K) > = 2.2.

Locating densely connected subnetworks (organizers) of Transcription Factors

In the network of TFs (nodes: Transcription Factors, links: regulatory interactions) we identified subnetworks distinguished by their dense interconnection and central role (i.e., organizers) by using an iterative layer-peeling algorithm [6], as follows. After first removing all autoregulatory loops, we repeatedly removed the nodes in the top and bottom layers of the network until only three small isolated (graph) components ('cores') remained. To these cores we then added in 3 subsequent steps their up- and downstream intermediate regulators to obtain three major organizers (see Results).

Alternatively, to locate overlapping, densely connected groups of nodes among the 69 non-isolated TFs we applied CFinder [7] to the underlying undirected network and identified the *k*-clique communities (groups of densely interconnected nodes) at *k* = 3 corresponding to 'rolling' a triangle by moving one of its nodes at each step.. Note that any TF (node) was allowed to belong to more than one community. Next, we added to each community, C_A, all nodes reachable from a node of C_A via regulatory interactions, but not yet contained by any of the communities. Last, we merged communities C_A and C_B, if all exclusively contained nodes of C_A were directly regulated by an exclusively contained node of C_B.

Significance of the transcriptional response of a group of genes

Our goal was to quantify the effect of particular (environmental or internal) conditions (or signals) *S* on the transcript levels of a selected group of genes. First, we grouped experiments (GSMs, Geo SaMples) according to their platforms (GPLs). Then to each experiment obtained under a 'normal' condition (*e.g.*, stationary state) we assigned the signal $S=-1$ and to all others (*e.g.*, hyper-osmotic shock, N depletion, or DNA damage with MMS) we assigned the signal $S=+1$. Next, we computed the Pearson correlation, C_i, between the *i*th gene's expression E_{ij} and the jth experimental condition S_j. using

$$C_i\left(E_{ij}, S_j\right) = \frac{\left\langle \mathrm{E_{ij} S_j} \right\rangle_j - \left\langle E_{ij} \right\rangle_j \left\langle S_j \right\rangle}{\left[\left\langle E_{ij}^2 \right\rangle_j - \left\langle E_{ij} \right\rangle_j^2\right]^{1/2} \left[1 - \left\langle S_j \right\rangle^2\right]^{1/2}},$$

where the subscript *j* includes both those experiments under the condition of interest (i.e. experiments a_1, a_2, …, a_n, signal value: $S_j = +1$) and those under 'normal' conditions ($j=b_1$, b_2, …, b_m, and $S_j = -1$). The *i*th gene's response to signal *S* is significant, *i.e.*, it is strongly activated (repressed), if its C_i value is higher (lower) than the majority of the correlation values calculated for all yeast genes. This can be measured with the Z score, $\mathrm{Z_i} = \left|\mathrm{C_i} - \mathrm{C}\right| / \Delta\mathrm{C}$, of the *i*th gene's

response, where *C* and *ΔC* are the average and standard deviation of the correlation values of all yeast genes. Here we use the absolute value, because a strong activation and a strong repression are equally important responses and should both give a high Z score.

The significance of the response of the entire group *G* to condition *S* can be assessed by comparing the average *Z* score in *G*, $Z_G = \langle Z_i \rangle_{i \in G}$, to the similarly computed averages (Z_{H1}, Z_{H2} ,…) in other, randomly selected groups of genes of the same size (*H1*, *H2*, …). We used 1,000 such control groups. Denoting by $\langle Z_H \rangle$ and ΔZ_H the average and standard deviation of Z_H values, the double Z score of the response of group *G* is $Y_G = (Z_G - \langle Z_H \rangle)/\Delta Z_H$.

Supplementary Table

Motif conserved in	at least 2 other yeast		at least 1 other yeast		(no criteria)	
**P value threshold**	**< 0.001**	**< 0.005**	**< 0.001**	**< 0.005**	**< 0.001**	**< 0.005**
**# of nodes**	1 905	2 323	2 277	2 815	2 883	3 663
input	54	48	50	40	37	23
intermediate	45	53	50	61	65	79
regulator (in.+int.)	99	101	100	101	102	102
output	1 806	2 222	2 177	2 714	2 781	3 561
**# of links**	3 406	4 890	4 440	6 674	6 448	10 592
autoregulatory	12	15	13	16	16	20

Table S1. Statistics of the six available versions of the Harbison et.al data set.

Data was downloaded from http://jura.wi.mit.edu/fraenkel/download/release_v24/. The 6 versions differ in the confidence level of TF binding based on microarray data (p value < 0.001 or p value < 0.005) and the level of evolutionary conservation of a DNA binding sequence motif (conserved in at least 2, 1 or 0 other closely-related yeast species). For the analyses in the main text of the paper the most stringent version of the data set was used (P < 0.001, conservation in at least 2 other yeast strains).

Supplementary Figures

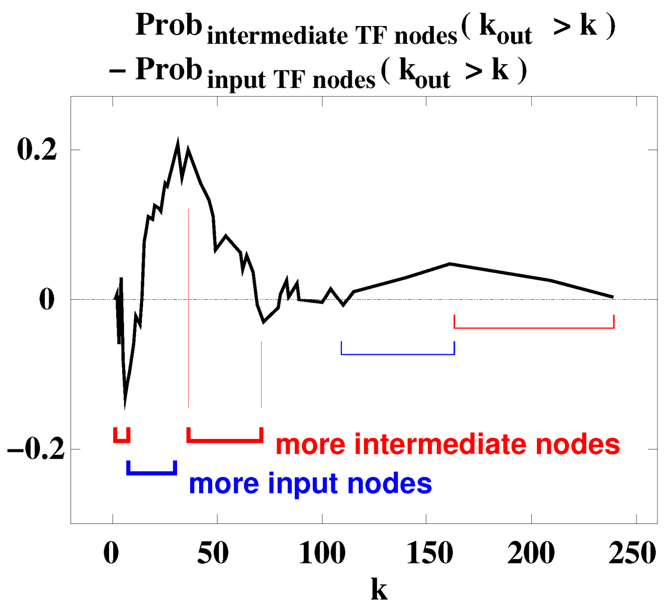

Figure S1. Centrally located (intermediate TF) nodes of the network tend to have a larger number of outgoing connections.

Centrally located (intermediate TF) nodes of the network tend to have a larger number of outgoing connections than less centrally located (input TF) nodes. This is in contrast to certain man-made communication networks, such as the Internet, where centrally located nodes in a network often have a small number of connections [8,9]. Note that in this graph the difference of two probabilities is plotted, where both of these probabilities is the negative integral of the corresponding density function. Thus, a significant positive slope in this graph indicates the dominance of input (intermediate) nodes.

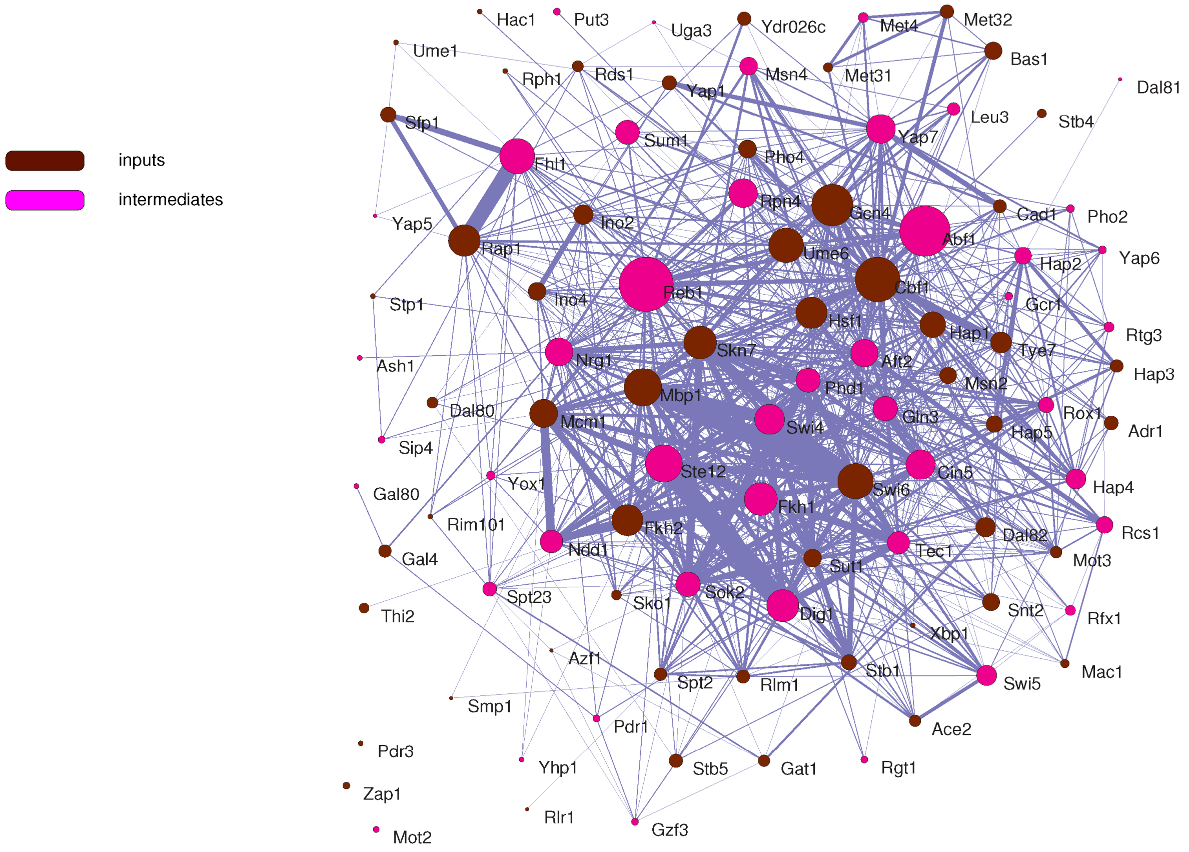

Figure S2A. Enlarged Figure 2*A*.

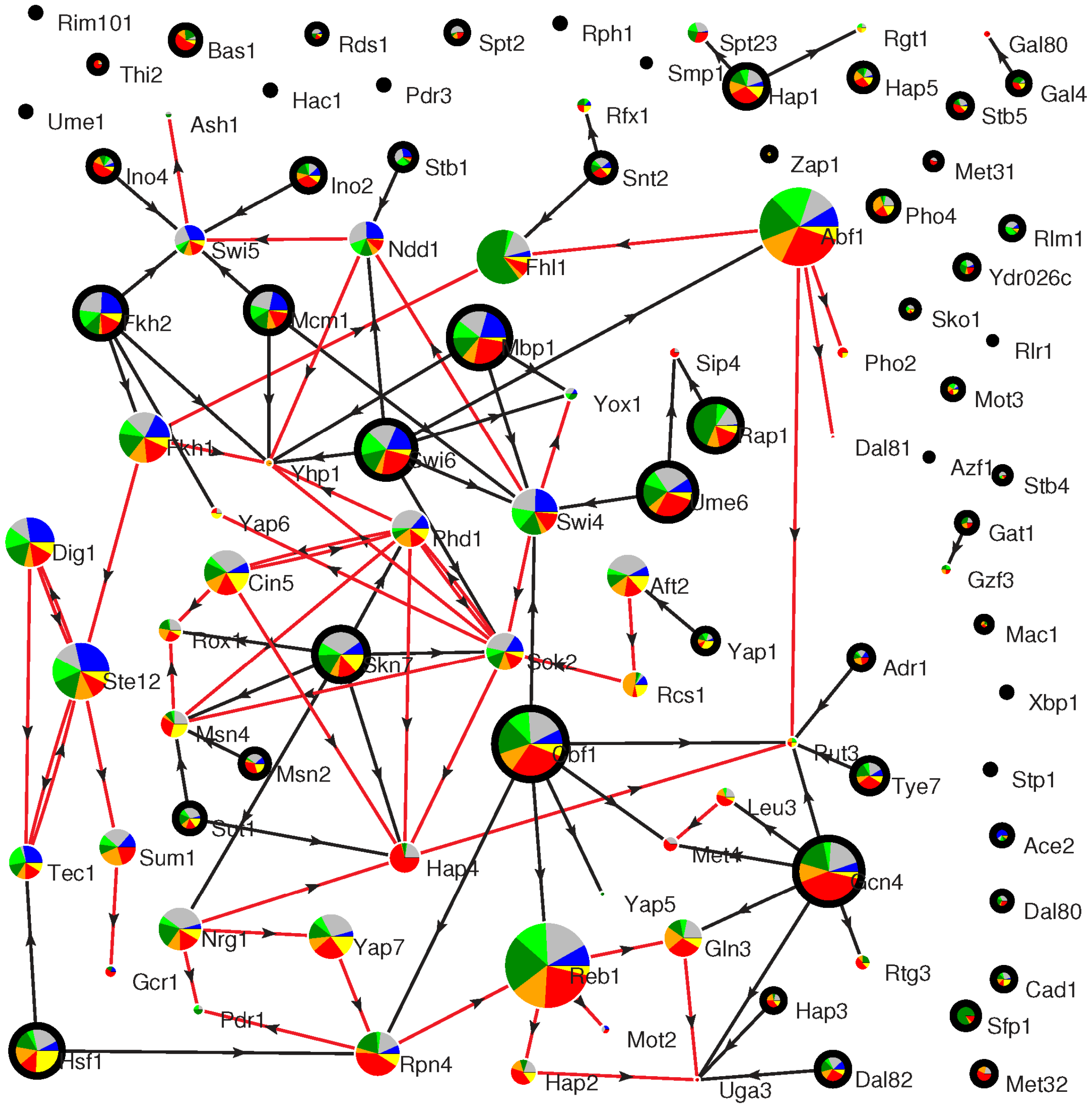

Figure S2B. Enlarged Figure 2*B*.

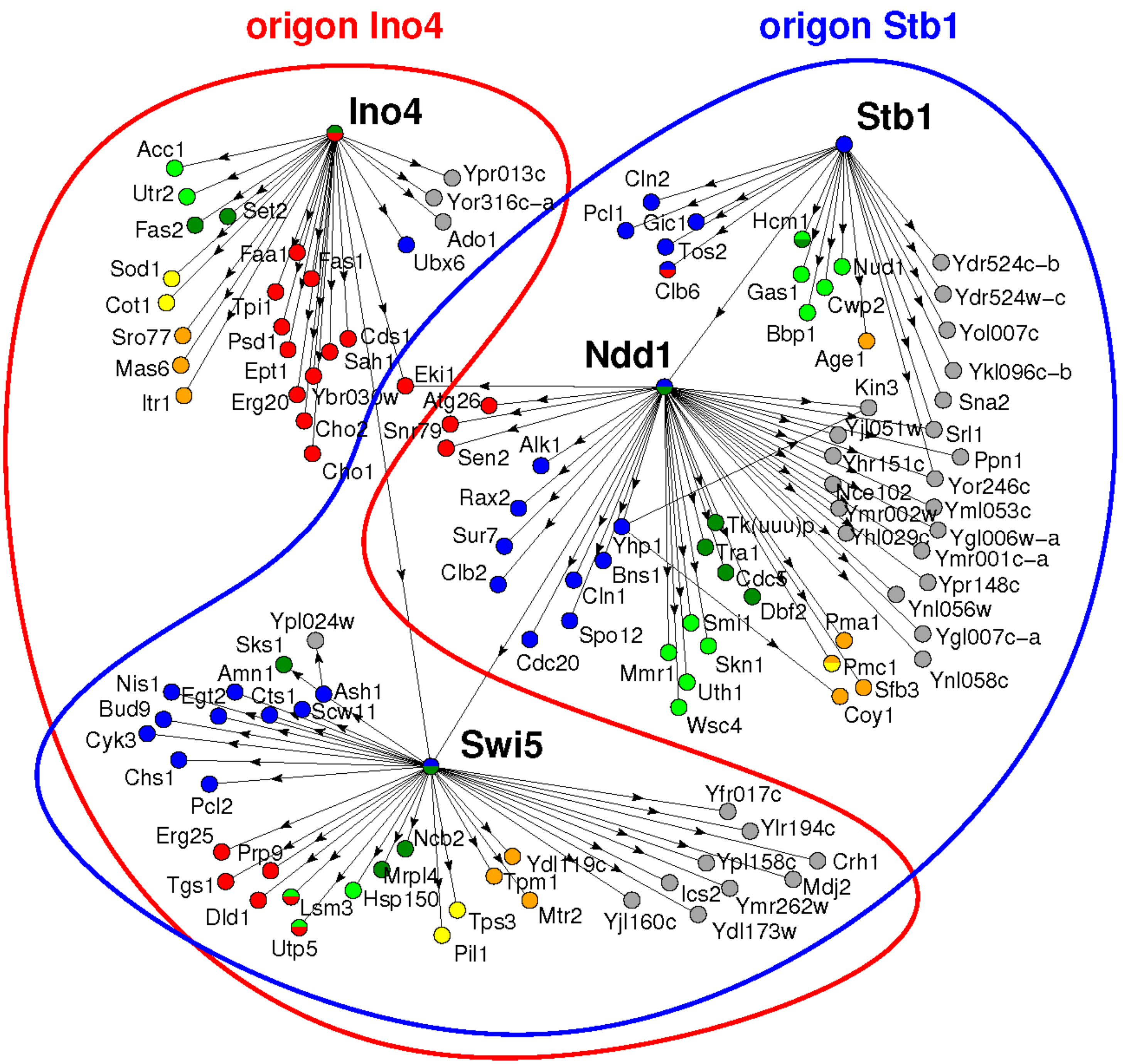

Figure S2C. Enlarged Figure 2*C*.

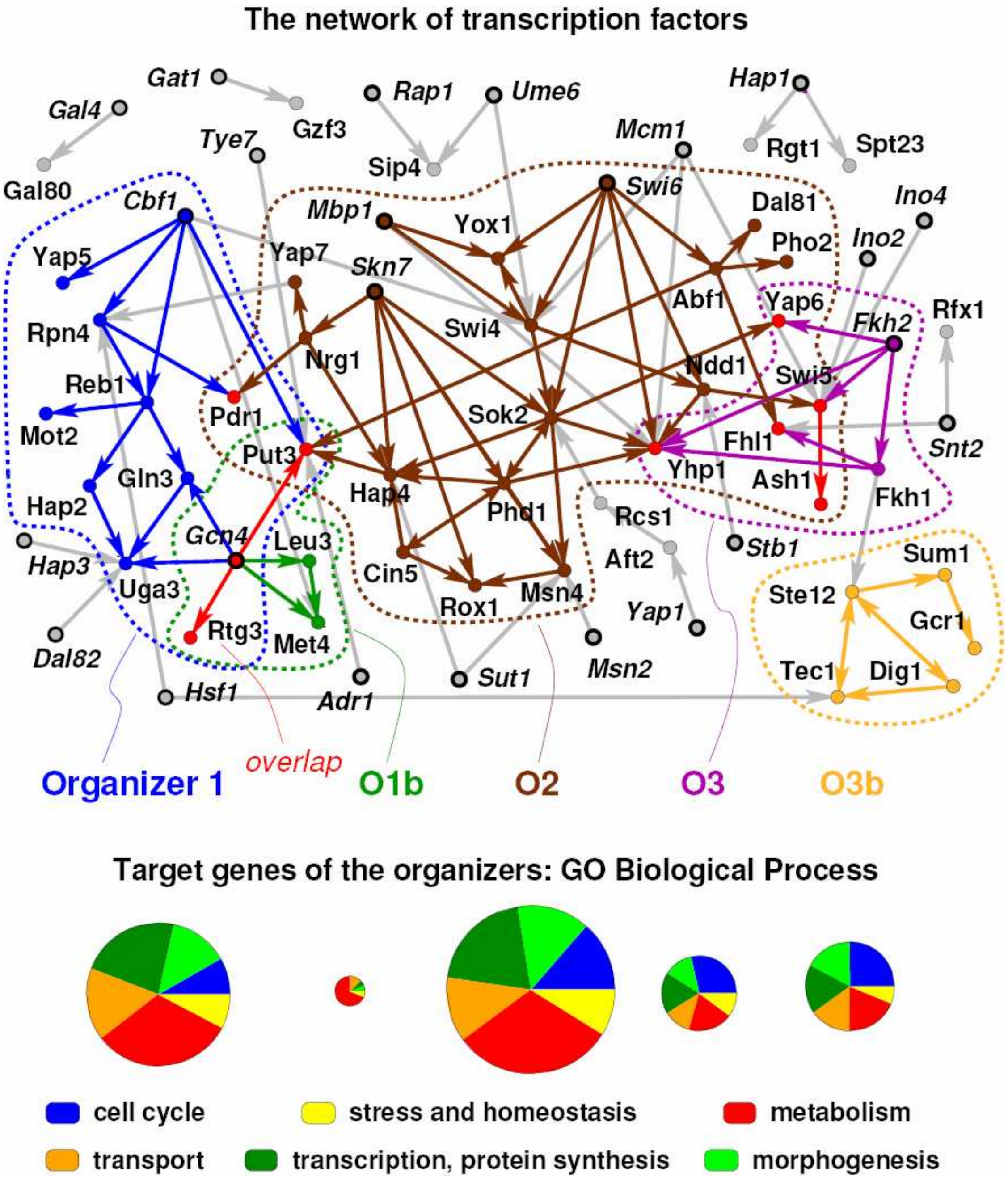

Figure S3. Identification of communities within the *S. cerevisiae* TR network

Top panel: The network of transcription factors (TFs) in *S. cerevisiae* (using the highest stringency (A) data set; P<0.001 and conservation in 2 closely related yeast species) Each link (node) represents a regulatory interaction (a TF protein and its gene). Auto-regulatory interactions and isolated nodes are not shown. The internally densely connected groups of TFs in the TR network (organizers,

see Methods) are colored blue, green, brown, magenta and yellow. Nodes and links belonging to more than one group (i.e., overlaps) are red. Input TFs are marked. *Bottom panel*: GO Slim Biological Process functions in the organizers. For each organizer the TFs contained only by this organizer plus their target genes were listed. The total size of a pie is proportional to the number of genes listed for the organizer and the size of a pie section is proportional to the number of times that the according term annotates these genes (see Methods for details). Observe that in Organizers 1, 2 and especially 1b metabolism (red) is overrepresented, while organizers 3 and 3b have a relatively higher content of cell-cycle related functions (blue).

P value threshold	P < 0.001			P < 0.005		
Conserved in at least # other yeast	2	1	0	2	1	0
Figure #	A	B	C	D	E	F

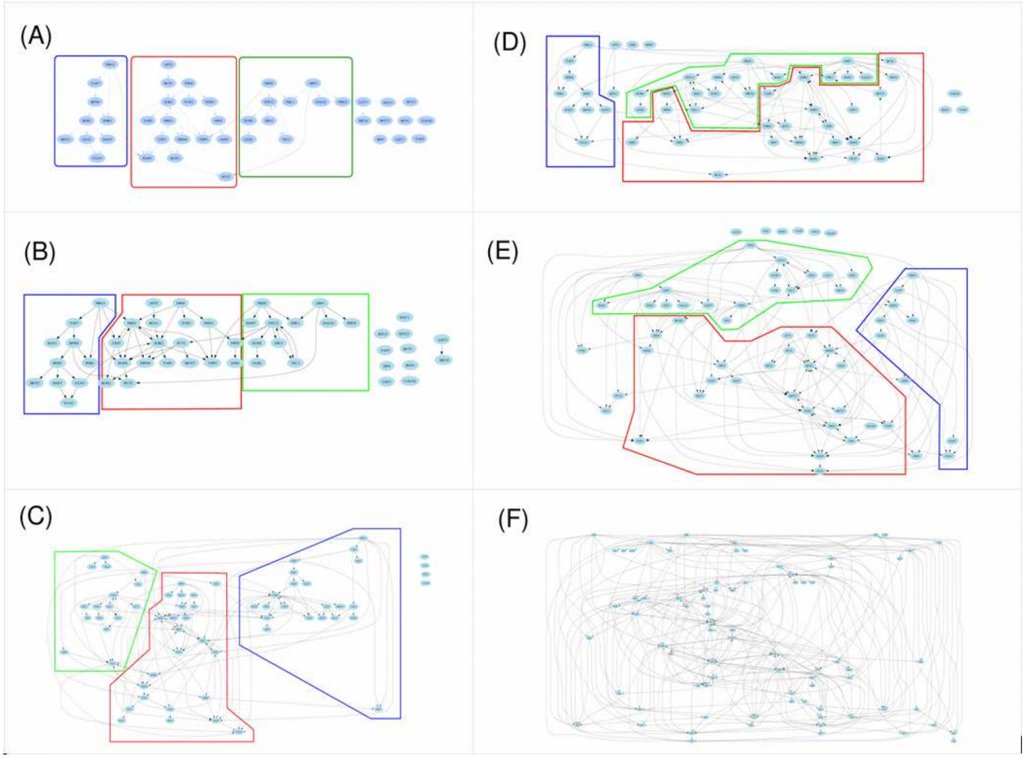

Figure S4. Organizers using the six different versions of the Harbison et.al. data set.

The three densely connected subnetworks (organizers) of the yeast TR network are enclosed by blue, red, and green rectangles, respectively, while non-connected intermediate TFs are at the right. Note, that while the organizers are apparent in the highest (A) and relatively high stringency datasets (B-D), the organizers are less apparent in the low stringency data (E) and essentially unrecognizable in the lowest stringency data (F). Note, that TFs of organizers of dataset A are exact subsets of those of dataset B, dataset B of dataset C, etc. The only discrepancy is in dataset D and E where one of the TF nodes is catalogued into another organizer.

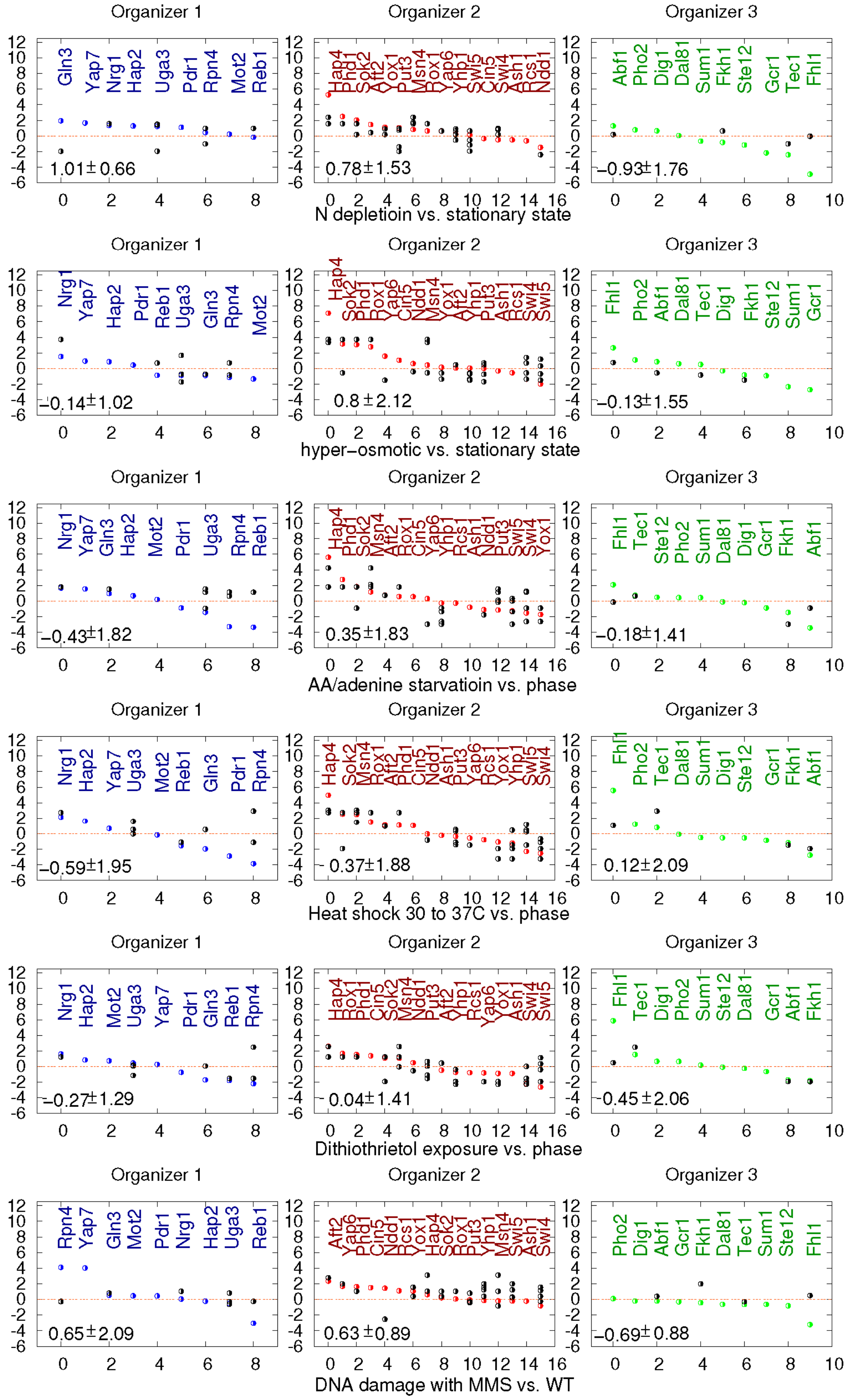
Organizer 1
Organizer 2
Organizer 3
Gln3 Yap7 Nrg1 Hap2 Uga3 Pdr1 Rpn4 Mot2 Reb1
Abf1 Pho2 Dig1 Dal81 Sum1 Fkh1 Ste12 Gcr1 Tec1 Fhl1
1.01±0.66
0.78±1.53
−0.93±1.76
N depletioin vs. stationary state
Nrg1 Yap7 Hap2 Pdr1 Reb1 Uga3 Gln3 Rpn4 Mot2
Fhl1 Pho2 Abf1 Dal81 Tec1 Dig1 Fkh1 Ste12 Sum1 Gcr1
−0.14±1.02
0.8±2.12
−0.13±1.55
hyper−osmotic vs. stationary state
Nrg1 Yap7 Gln3 Hap2 Mot2 Pdr1 Uga3 Rpn4 Reb1
Fhl1 Tec1 Ste12 Pho2 Sum1 Dal81 Dig1 Gcr1 Fkh1 Abf1
−0.43±1.82
0.35±1.83
−0.18±1.41
AA/adenine starvatioin vs. phase
Nrg1 Hap2 Yap7 Uga3 Mot2 Reb1 Gln3 Pdr1 Rpn4
Fhl1 Pho2 Tec1 Dal81 Sum1 Dig1 Ste12 Gcr1 Fkh1 Abf1
−0.59±1.95
0.37±1.88
0.12±2.09
Heat shock 30 to 37C vs. phase
Nrg1 Hap2 Mot2 Uga3 Yap7 Pdr1 Gln3 Reb1 Rpn4
Fhl1 Tec1 Dig1 Pho2 Sum1 Ste12 Dal81 Gcr1 Abf1 Fkh1
−0.27±1.29
0.04±1.41
−0.45±2.06
Dithiothrietol exposure vs. phase
Rpn4 Yap7 Gln3 Mot2 Pdr1 Nrg1 Hap2 Uga3 Reb1
Pho2 Dig1 Abf1 Gcr1 Fkh1 Dal81 Tec1 Sum1 Ste12 Fhl1
0.65±2.09
0.63±0.89
−0.69±0.88
DNA damage with MMS vs. WT

Figure S5. Node-signal covariance of organizers.

Transcriptional responses of the target genes of the organizer intermediate TFs in response to six external conditions (one row for each condition). The double Z scores (Y axis) measure the significance of the response of the organizer node and its target genes to an external condition as compared to a control condition. Points are colored according the organizer they belong to (blue, red and green) and black points show the regulator (an input TF) of each intermediate TF. In each subfigure along the X axis the TFs of the organizer are listed. The numbers in the bottom part of each graph denote the average double Z scores for O1 (blue) O2 (red) and O3 (green), respectively, while the colored dots represent the average double Z-score of genes regulated by the indicated intermediate TF. Black dots represent the same for the input TF regulating the indicated intermediate TF. The difference between inputs and intermediates suggests that the signal-covariance of the organizers are less affected by their inputs and more by the topology of the organizers.

References

1. Harbison CT, Gordon DB, Lee TI, Rinaldi NJ, Macisaac KD, Danford TW, Hannett NM, Tagne JB, Reynolds DB, Yoo J, Jennings EG, Zeitlinger J, Pokholok DK, Kellis M, Rolfe PA, Takusagawa KT, Lander ES, Gifford DK, Fraenkel E, Young RA: **Transcriptional regulatory code of a eukaryotic genome.** *Nature* 2004, 431: 99-104
2. Batagelj V, Mrvar A: **PAJEK -- Program for large network analysis.** *Connections* 1998, 21: 47-57
3. Milo R, Shen-Orr S, Itzkovitz S, Kashtan N, Chklovskii D, Alon U: **Network motifs: simple building blocks of complex networks.** Science 2002, 298: 824-827
4. Martin D, Brun C, Remy E, Mouren P, Thieffry D, Jacq B: **GOToolBox: functional analysis of gene datasets based on Gene Ontology.** *Genome Biol.* 2004, 5: R101
5. Balazsi G, Barabasi AL, Oltvai ZN: **Topological units of environmental signal processing in the transcriptional regulatory network of Escherichia coli.** *Proc Natl Acad Sci U S A* 2005, 102: 7841-7846
6. Wuchty S, Almaas E: **Peeling the yeast protein network.** *Proteomics* 2005, 5: 444-449
7. Adamcsek B, Palla G, Farkas IJ, Derenyi I, Vicsek T: **CFinder: locating cliques and overlapping modules in biological networks.** *Bioinformatics* 2006, 22: 1021-1023
8. Pastor-Satorras R, Vespignani A: *Evolution and Structure of the Internet.* Cambridge University Press; 2004
9. Doyle JC, Alderson DL, Li L, Low S, Roughan M, Shalunov S, Tanaka R, Willinger W: **The "robust yet fragile" nature of the Internet.** *Proc Natl Acad Sci U S A* 2005, 102: 14497-14502
